# Dipyridamole-Coated 3D-Printed β-Tricalcium Phosphate Scaffolds: Spectrophotometric Characterization, Drug Release Kinetics, and In Vitro Evaluation to Guide Critical-Sized Bone Defect Repair Studies

**DOI:** 10.3390/jfb17080396

**Published:** 2026-08-11

**Authors:** Purva Rasane, Vasudev Vivekanand Nayak, Lahiru Chamara Weerasinghe Arachchige, Eleni Rice, Zeinab Fotouhi Ashin, Bharath Venkatesan, Venu Varanasi, Noriaki Ono, Simon Young, Lukasz Witek

**Affiliations:** 1 Biomaterials and Regenerative Biology Division, NYU College of Dentistry, New York, NY 10010, USA; 2 Department of Biochemistry and Molecular Biology, University of Miami Miller School of Medicine, Miami, FL 33136, USA; 3 Dr. John T. Macdonald Foundation Biomedical Nanotechnology Institute (BioNIUM), University of Miami, Miami, FL 33136, USA; 4 Institut de Biologie Valrose (iBV), Université Côte d’Azur, Parc Valrose, 06107 Nice, France; 5 Department of Biomedical Engineering, NYU Tandon School of Engineering, Brooklyn, NY 11201, USA; 6 Bone-Muscle Research Center, College of Nursing and Health Innovation, The University of Texas at Arlington, Arlington, TX 76019, USA; 7 Department of Diagnostic and Biomedical Sciences, The University of Texas Health Science Center at Houston (UTHealth Houston) School of Dentistry, Houston, TX 77054, USA; 8 Katz Department of Oral and Maxillofacial Surgery, The University of Texas Health Science Center at Houston (UTHealth Houston) School of Dentistry, Houston, TX 77054, USA; 9 Hansjörg Wyss Department of Plastic Surgery, NYU Grossman School of Medicine, New York, NY 10016, USA; 10 Department of Oral and Maxillofacial Surgery, NYU College of Dentistry, New York, NY 10010, USA

**Keywords:** 3D-printing, β-tricalcium phosphate, dipyridamole, bone tissue engineering, critical-sized bone defects, drug release kinetics, physicochemical characterization

## Abstract

Critical-sized bone defects remain a significant clinical challenge, and dipyridamole (DIPY)-coated 3D-tricalcium phosphate (β-TCP) scaffolds have shown promising osteogenic efficacy in preclinical models. However, the literature on the systematic physicochemical characterization of this scaffold system, including optimization of DIPY loading parameters, release kinetics, and surface properties, is lacking. This study addresses these gaps by characterizing DIPY-loaded 3D-printed β-TCP scaffolds across solid and porous architectures, three coating concentrations (10, 100, and 1000 µM), and three coating volumes (250, 500, and 1000 µL). Under static PBS conditions, drug release over 21 days was quantifiable only at 1000 µM, and release-kinetics modeling (zero-order, Higuchi, and Korsmeyer–Peppas) was therefore restricted to this highest concentration. At 1000 µM, both scaffold types showed biphasic release profiles, with standard empirical models reasonably approximating the overall kinetics, while not fully capturing the biphasic behavior over the entire duration. Porous scaffolds showed significant volume-dependent release (*p* = 0.002, *η*^2^ = 0.88), attributable to drug penetration into the interconnected macropore network, whereas solid scaffolds displayed volume-independent release confined to external surfaces. Scanning electron microscopy revealed concentration-dependent needle-shaped DIPY crystal deposition while contact angle measurements indicated no significant changes in surface hydrophilicity. MTT assay demonstrated biocompatibility at all concentrations, with viability influenced by coating volume rather than drug concentration. These findings establish a foundational physicochemical framework for DIPY-loaded β-TCP scaffolds, providing the baseline data necessary to guide subsequent biological evaluation and translational efforts toward critical-sized craniofacial and orthopedic bone defect repair.

## 1. Introduction

Critical-sized bone defects require therapeutic intervention to facilitate healing [1], and can arise from a range of etiologies, including high-impact trauma, chronic or aggressive infections, tumor enucleation, radiotherapy, and congenital anomalies [2]. Reconstruction in the oral and maxillofacial region is especially complex, due to the recurrent functional movement of the jaw, the exposure of surgical sites to the oral cavity or sinus, and diminished local blood supply [3,4,5,6]. These factors could collectively compromise the regenerative environment and increase the risk of wound dehiscence, infection, and graft failure [3,4,5,6]. Autologous bone grafting remains the clinical gold standard for reconstruction of large bone defects [7,8], yet is limited by drawbacks including the restricted availability and the risk of donor site morbidity [9,10]. These limitations have prompted the development of bone tissue engineering strategies using 3D-printed bioactive alloplastic scaffolds [11]. Among materials commonly used to synthesize these scaffolds, beta-tricalcium phosphate (β-TCP) has gained prominence as it closely mimics the mineral composition of bone. β-TCP has been shown to be biocompatible, osteoconductive, and bioresorbable with a favorable safety profile [12,13,14]. Three-dimensional (3D) printing has further enabled the production of β-TCP scaffolds with engineered pore architectures to modulate the degradation rate, promote vascular ingrowth, and improve mechanical stability, while being customized to match patient-specific defect geometry [15]. For example, our previous study on 3D-printed β-TCP scaffolds revealed a degradation rate of up to 90.5% per annum in a rabbit model of critical-sized alveolar bone defect healing [16].

Despite these benefits, uncoated β-TCP scaffolds fail to provide the desired osteogenic and angiogenic response [17]. Thus, these scaffolds are frequently coated with bioactive molecules such as recombinant human bone morphogenic proteins (rhBMPs) to augment their osteogenic capacity [18]. However, concerns regarding ectopic bone formation, risk of premature suture fusion, immunogenicity, and high cost of rhBMPs have motivated the search for alternative osteogenic agents [18,19,20,21]. Previous studies have shown that an antiplatelet and vasodilator, dipyridamole (DIPY), serves as an indirect adenosine receptor agonist (A_2_AR), and promotes bone regeneration by inhibiting cellular adenosine uptake via the Equilibrative Nucleoside Transporter [22,23]. A_2_AR activation suppresses inflammatory osteolysis and increases osteoblast numbers in inflamed bone, thereby promoting osteoblast expression of key markers for bone formation, such as osteocalcin, osteonectin, and alkaline phosphatase [24,25]. When applied as a coating on 3D-printed bioactive ceramic scaffolds, DIPY has demonstrated improved bone formation relative to uncoated scaffolds in axial and appendicular preclinical models [26,27,28,29]. Unlike rhBMP, DIPY has also been shown to augment bone repair without inducing ectopic bone formation or premature suture fusion in pediatric craniomaxillofacial defects, offering a safer pharmacological alternative to rhBMP [28,30].

However, as per the Biopharmaceutics Classification System (BCS), DIPY is classified as a Class II compound, characterized by low aqueous solubility and high permeability [31]. This compound demonstrates pH-dependent solubility with higher dissolution in acidic conditions, along with high solubility in polar organic solvents like dimethyl sulfoxide (DMSO) and ethanol [32]. DMSO is commonly employed as a solvent vehicle in bone-targeted drug delivery systems and has also been recently used as a DIPY carrier in pre-clinical translational work with no adverse effects [28,29,30]. While its poor aqueous solubility poses challenges for systemic delivery, this same property can be leveraged for surface adsorption-based scaffold coating, which involves evaporation of a polar organic solvent vehicle (the carrier) and subsequent DIPY precipitation and adsorption onto the biomaterial surface. Furthermore, coating parameters such as concentration and volume could be varied to tailor drug loading and subsequent release kinetics [33].

Despite DIPY’s ability to augment bone formation, previously published studies have employed empirically selected DIPY concentrations without detailed justification or optimization protocols. These studies have used concentrations ranging from 10 µM to 1000 µM without a standardized rationale [26,29,30]. This lack of standardization limits reproducibility, which may hinder translation toward clinical applications. To address this gap, the present study aimed to characterize the effects of varying DIPY concentrations and coating volumes on β-TCP scaffolds using UV–visible spectrophotometry to establish a standard curve and to evaluate DIPY release kinetics from the scaffold surface. By defining these foundational material properties, this work establishes the analytical framework necessary for the reproducible and evidence-based fabrication of bioactive bone scaffolds.

## 2. Materials and Methods

### 2.1. β-TCP Colloidal Gel Preparation and Scaffold Fabrication

**Figure 1:**
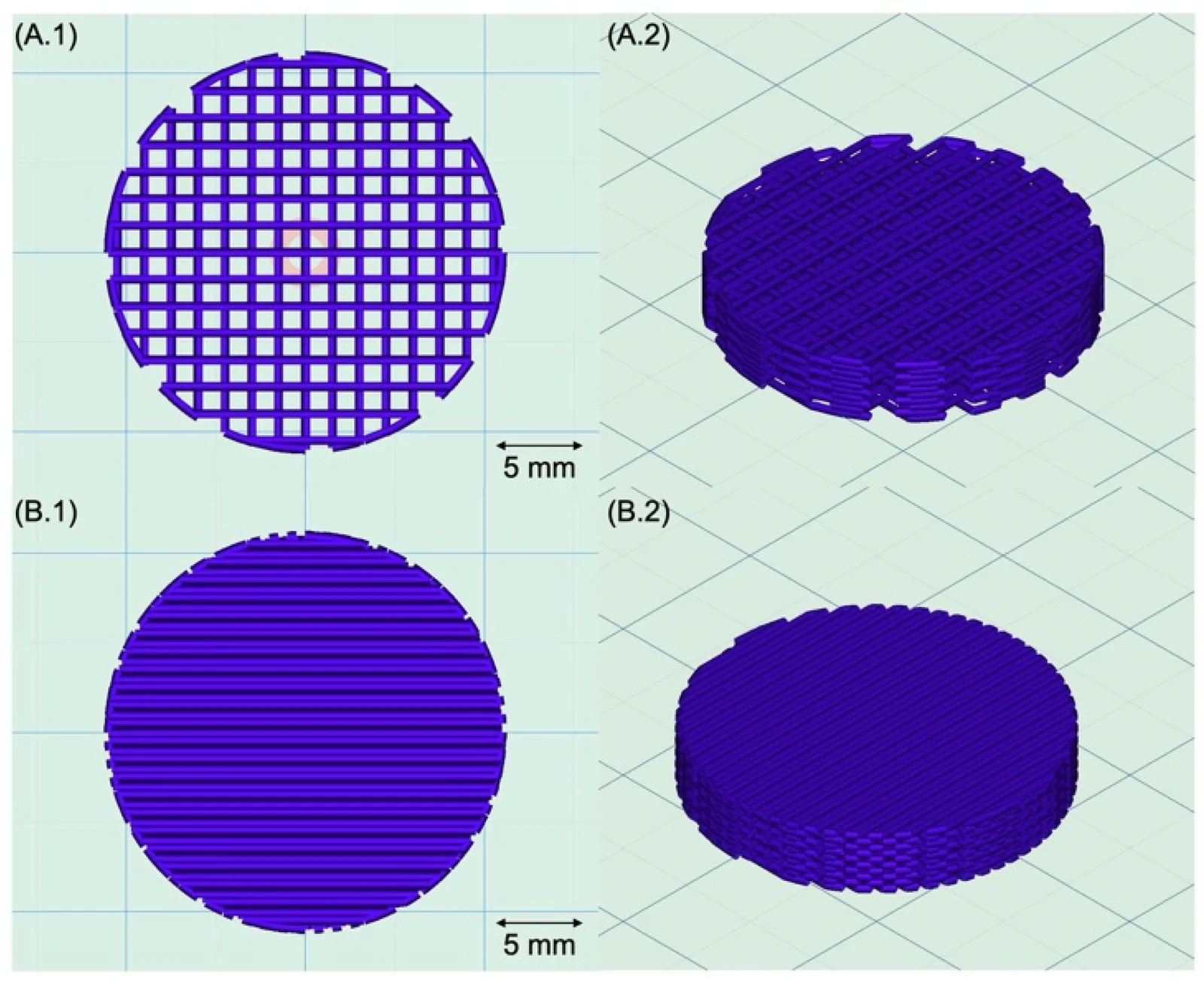
Computer-aided design (CAD) showing the top and isomeric views of the (**A.1**,**A.2**) porous, and (**B.1**,**B.2**) solid scaffolds.

A colloidal gel suspension was synthesized using calcined and milled β-TCP powder (ThermoFisher Scientific, Waltham, MA, USA) at a solid volume fraction of ~46%, as shown previously [30,34]. The prepared colloidal gel suspension was loaded into a Luer Lok syringe (Nordson, Westlake, OH, USA) fitted with a precision fluid dispensing tip with an outer diameter of 250 µm (Nordson, Westlake, OH, USA). Circular solid scaffolds measuring 2 mm in height and 11 mm in diameter were designed in RoboCAD v6 (3D Inks LLC, Tulsa, OK, USA) and fabricated using a custom-built direct inkjet write 3D printer (3D Inks LLC, Tulsa, OK, USA). Porous scaffolds were designed and printed to the same parameters as solid scaffolds. In the porous β-TCP scaffolds, 250 µm struts arranged with 500 µm inter-rod spacing produced a periodic, fully interconnected lattice of ~500 µm square macropores (Figure 1). Following printing, all scaffolds were sintered in a high-temperature furnace (LT 9/12, Nabertherm, Lilienthal, Germany) using a multi-stage thermal profile: an initial dwell at 400 °C to remove low-molecular-weight organic components, followed by a second dwell at 900 °C for pyrolysis of residual carbonaceous binder, and a final sintering stage at 1100 °C for 4 h to achieve solid-state densification of the ceramic matrix [34].

### 2.2. DIPY Solution Preparation and UV–Vis Spectrophotometry

A 0.1 M stock solution of DIPY was prepared in dimethyl sulfoxide (DMSO; Sigma-Aldrich, Burlington, MA, USA). A series of DIPY working solutions was prepared across a broad concentration range by diluting the stock solution with Phosphate-Buffered Saline (PBS 1×, pH 7.4; Gibco, ThermoFisher Scientific, Waltham, MA, USA). The final calibration curve was established using concentrations of 0, 10, 25, 50, 100, and 1000 µM as these points were within the instrument’s linear absorbance range (0–3.5 OD) and provided a good correlation (*R*^2^ > 0.999). These solutions were used to generate UV–Vis standard curves by measuring absorbance at 405 nm using a UV–Vis spectrophotometer (Molecular Devices Filtermax^TM^ F3/F5, San Jose, CA, USA). Standard curves were generated by plotting absorbance versus concentration, and linear regression analysis was performed. Based on the linearity of the standard curve, 3 DIPY concentrations (10, 100, and 1000 µM), corresponding to DMSO contents of 0.01%, 0.1%, and 1% *v*/*v*, respectively, were selected to span a broad, 100-fold logarithmic range for scaffold coating experiments. Although residual DMSO was not directly quantified, this coating protocol is consistent with previously reported DIPY-augmented 3D-printed bioactive ceramic scaffolds, in which β-TCP constructs were coated or immersed in DMSO-based DIPY solutions and implanted into craniofacial and long bone defect models without a dedicated solvent removal step, yet demonstrated enhanced bone regeneration and no histologic evidence of local inflammation or toxicity [28,29].

### 2.3. DIPY Coating Methodology

Both porous and solid scaffolds were coated using a drop-casting approach in which DIPY solutions were pipetted onto the scaffold and allowed to dry by solvent evaporation, similar to solution-dropping methods commonly used to load pharmaceutical compounds onto 3D-printed scaffolds [35,36,37]. Each scaffold was coated at 3 DIPY concentrations (10, 100, and 1000 µM, prepared from a 0.1 M DMSO stock diluted in PBS) and 3 total volumes (250, 500, and 1000 µL), with PBS-coated scaffolds serving as controls. For porous scaffolds, samples were placed in the wells of a 48-well plate, and the allocated volume was added until the scaffold was fully immersed. Because of the interconnected pore architecture, the coating solution could not be constrained to a single surface and therefore infiltrated the entire pore network. For solid scaffolds, a 3D-printed custom device was used to apply the coating bilaterally. In brief, for a given total volume, half was deposited on one face and allowed to evaporate at 37 °C for at least 24 h, after which the scaffolds were inverted and the remaining half volume applied to the opposite face and dried under the same conditions. This strategy was chosen to confine DIPY primarily to the external surfaces of non-porous scaffolds, provide symmetric exposure from both faces in release and cell-culture experiments, and allow direct calculation of the theoretical drug mass per scaffold based on known coating volume and concentration, rather than to enforce uniform drug distribution throughout the scaffold interior.

### 2.4. Quantification of Drug Loading

The theoretical drug mass for each scaffold was calculated from the applied concentration and volume, assuming minimal loss to the surrounding environment. These values served as reference estimates for total drug loading per scaffold. For mass conversion, the molecular weight of DIPY (504.6 g/mol) was used, and the drug amount loaded was determined as:

(1) $$Drug\;Mass\;(µg)=\frac{Drug\;Amount\;(nmol)\times 504.6\;(g\cdot {mol}^{-1})}{10^{-3}}$$

The total drug mass loaded per scaffold for all coating conditions is summarized in Table 1.

### 2.5. Drug Release Studies

DIPY release was assessed under static conditions at 37 °C. Coated porous and solid scaffolds were individually immersed in 1 mL of 1× PBS in separate wells of a 48-well tissue culture plate. Aliquots were collected at predetermined time points spanning early intervals (0.5, 1, 2, 4, 6, and 8 h) and extended intervals (24 h, 7, 14, and 22 days). At each time point, the entire aliquot was withdrawn and replaced with an equal volume of fresh PBS to maintain sink conditions. Absorbance of collected samples was measured at 405 nm using a UV–Vis spectrophotometer (Molecular Devices FilterMax™ F3/F5, San Jose, CA, USA). DIPY concentration in each sample was determined by interpolation from the previously established standard curve. Cumulative drug mass released (µg) was calculated and plotted against time to generate release profiles for both solid and porous scaffold architectures.

### 2.6. Cell Culture and Scaffold Preparation

Human embryonic stem cells (RUES2), initially maintained under standard feeder-free pluripotent culture conditions, were induced toward a mesenchymal-like phenotype using a serum-containing Dulbecco’s Modified Eagle’s Medium (DMEM)-based medium adapted from a previously reported embryoid body-independent differentiation protocol [38]. The RUES2 cells were provided by Dr. Edwin A. Rosado-Olivieri and the Laboratory of Human Organogenesis, Department of Molecular Pathobiology, NYU College of Dentistry. Cells were dissociated with Accutase (STEMCELL Technologies, Vancouver, BC, Canada) and transferred to high-glucose DMEM (Gibco™, ThermoFisher Scientific, Waltham, MA, USA) supplemented with 10% fetal bovine serum (FBS; EmbryoMax ES cell-qualified, Millipore Sigma, St. Louis, MO, USA), 1 mM sodium pyruvate (Gibco™, ThermoFisher Scientific, Waltham, MA, USA), and 1% antibiotic–antimycotic solution (Gibco™, Thermo Fisher Scientific, Waltham, MA, USA). Rho-associated kinase (ROCK) inhibitor (Y-27632, 10 µM; Tocris, Bristol, UK) was included for the first 24 h after seeding to enhance cell survival, after which the medium was replaced with ROCK inhibitor-free medium. Cells were expanded to approximately 80% confluence and subcultured using 0.05% trypsin–EDTA (Gibco™, Thermo Fisher Scientific, Waltham, MA, USA). For scaffold seeding, ROCK inhibitor was again added for the first 24 h following cell attachment and subsequently removed by medium replacement. For in vitro studies, β-TCP scaffolds were sterilized by autoclaving at 121 °C before DIPY coating. Scaffolds were subsequently coated with DIPY at concentrations of 10, 100, or 1000 µM, or with 1× PBS as a vehicle control, each applied at coating volumes of 250, 500, or 1000 µL. Coated scaffolds were transferred to a 37 °C oven and allowed to dry until solvent evaporation was achieved (approximately 72 h).

### 2.7. Cell Viability Assessment

Cell viability was assessed using the 3-(4,5-dimethylthiazol-2-yl)-2,5-diphenyltetrazolium bromide (MTT, ThermoFisher Scientific, Waltham, MA, USA) assay at a pre-defined early time point (day 1). This early assessment served to rapidly screen the coating conditions and determine which combinations supported adequate cell metabolic activity without cytotoxicity. Sterilized scaffolds were placed individually in wells of a 48-well culture plate. A density of 11,000 cells per scaffold was used in 300 µL of growth medium and incubated under standard culture conditions (37 °C, 5% CO_2_, saturated humidity) for 24 h. After 24 h, the medium was aspirated and replaced with 300 µL per well of MTT working solution (0.5 mg/mL in growth medium). Plates were incubated for 3 h under the same conditions to allow viable cells to convert the MTT tetrazolium salt to insoluble formazan crystals. The MTT solution was then carefully removed, and formazan crystals were solubilized by adding 300 µL of DMSO to each well. Absorbance was measured at 595 nm using a multimode microplate reader (Molecular Devices Filtermax^TM^ F3/F5, San Jose, CA, USA). Absorbance values of all experimental groups were normalized using the absorbance values obtained for the PBS-coated scaffold group.

### 2.8. Surface Characterization

Surface characterization was conducted to evaluate scaffold morphology, drug crystal deposition, and wetting behavior. Qualitative visualization of surface microstructure and DIPY crystal presence across porous and solid scaffold architectures was performed by scanning electron microscopy (SEM; TM4000 Plus II, Hitachi, Tokyo, Japan) at an accelerating voltage of 10 kV. Wetting behavior on DIPY-coated and uncoated solid β-TCP scaffold surfaces was assessed by contact angle goniometry (SCA30, DataPhysics Instruments USA Corp., Charlotte, NC, USA) using 3 probe liquids, namely, deionized water, diiodomethane, and ethylene glycol—with 3 µL droplets deposited at 1 µL/s. Total surface energy (γ_total_) and its dispersive (γ_d_) and polar (γ_p_) components were calculated from the contact angle data using the Owens–Wendt–Rabel–Kaelble (OWRK) method [39].

### 2.9. Statistical Analysis

Statistical analyses were performed using SPSS (v31, IBM Corp., Armonk, NY, USA). Descriptive data are reported as mean ± standard error of the mean, unless otherwise specified. All experiments were performed with a sample size of *n* = 3 per group. The UV–Vis standard calibration curve was evaluated by linear regression of absorbance versus concentration, with the coefficient of determination (*R*^2^) used to confirm linearity and goodness of fit. Drug release data was analyzed using repeated-measures ANOVA with time as the within-subjects factor and coating volume as the between-subjects factor. For this analysis, the Greenhouse–Geisser correction was applied when the assumption of sphericity was violated. Pairwise comparisons were conducted with Bonferroni adjustment. Cell viability data from the MTT assay was analyzed using two-way ANOVA with coating volume and DIPY concentration as fixed factors, followed by Tukey HSD post hoc comparisons. Surface energy data derived from contact angle measurements were compared across coating groups using one-way ANOVA. For this analysis, Levene’s test was used to assess homogeneity of variances, and a Welch ANOVA was performed as an alternative when equal variances could not be assumed. Drug release kinetics were evaluated by fitting cumulative mass released to zero-order (cumulative release vs. time), Higuchi (cumulative release vs. square root of time), and Korsmeyer–Peppas (log-transformed fractional release vs. log-transformed time) models using linear regression, with *R^2^* used to assess goodness of fit. For all tests, statistical significance was set at α = 0.05, and effect sizes are reported as partial eta-squared (*η*^2^), where applicable.

## 3. Results

### 3.1. DIPY Standard Calibration Curve

DIPY calibration standards (0, 10, 25, 50, 100, and 1000 µM) were measured by UV–Vis spectrophotometry at 405 nm. The plot of absorbance versus DIPY concentration showed a linear relationship over this range, with the regression equation A = 0.0008C + 0.0297 and a correlation coefficient *R*^2^ = 0.9999, consistent with Beer–Lambert behavior for quantitative analysis of DIPY in subsequent samples (Figure S1) [40].

### 3.2. Drug Release Tests

**Figure 2:**
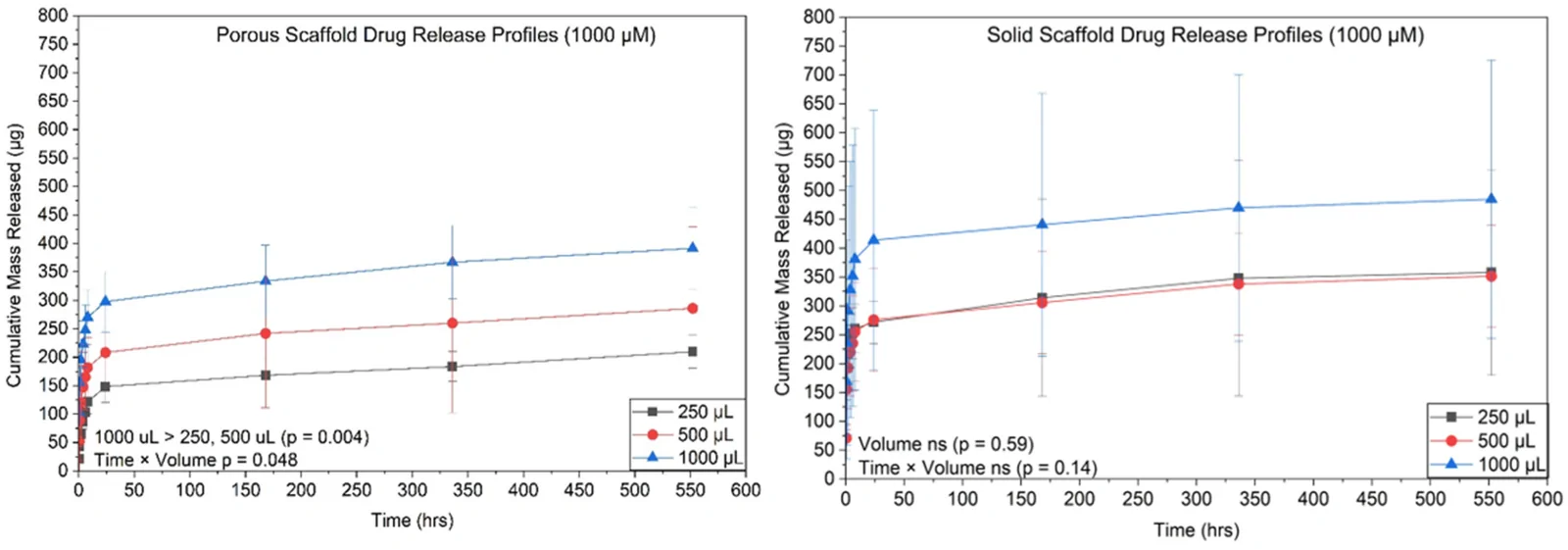
Cumulative DIPY release from porous and solid β-TCP scaffolds at 1000 µM for three coating volumes (250, 500, 1000 µL). Data is presented as mean ± standard deviation (*n* = 3 per coating volume group) over 21 days. For porous scaffolds, ANOVA indicates a significant main effect of coating volume (*p* = 0.002); Bonferroni-adjusted post-hoc comparisons showed greater cumulative release from the 1000 µL group than from the 250 and 500 µL groups (*p* = 0.004). The time × volume interaction was also significant (*p* = 0.048). For solid scaffolds, both the main effect of volume (*p* = 0.59) and the time × volume interaction (*p* = 0.14) are not significant.

The corrected absorbance values obtained for 10 µM and 100 µM were below detectable levels; therefore, only the 1000 µM group stratified across the 3 coating volumes, i.e., 250 µL, 500 µL, and 1000 µL, was considered for analysis. Cumulative DIPY release increased markedly over time for both porous and solid β-TCP scaffolds (porous: Greenhouse–Geisser (F(1.19,7.16) = 253.54, *p* < 0.001, partial *η*^2^ = 0.98; solid: F(1.36,8.15) = 112.41, *p* < 0.001, partial *η*^2^ = 0.95) (Figure 2). For porous scaffolds, coating volume exerted a strong effect on the time-averaged response (F(2,6) = 21.51, *p* = 0.002, partial *η*^2^ = 0.88). Bonferroni-adjusted post-hoc comparisons showed that the 1000 µL group exhibited significantly greater cumulative release than the lower coating volume groups (250 µL and 500 µL; *p* = 0.004), whereas the 250 µL and 500 µL groups did not differ (*p* = 1.000). The time and volume interaction values were also significant for porous constructs (F(2.39,7.16) = 4.62, *p* = 0.048, partial *η*^2^ = 0.61), indicating that release profiles over time depended on coating volume. In contrast, for solid scaffolds, the main effect of volume was not significant (F(2,6) = 0.57, *p* = 0.592, partial *η*^2^ = 0.16), and Bonferroni adjusted post-hoc comparisons revealed no differences between coating volumes (all *p* = 1.000), consistent with a non-significant time and volume interaction (F(2.72,8.15) = 2.49, *p* = 0.136, partial *η*^2^ = 0.45). Fractional degrees of freedom arise from the Greenhouse–Geisser correction applied to account for sphericity violations in the repeated-measures factors.

### 3.3. DIPY Pharmacokinetics

To characterize DIPY release from the coated β-TCP scaffolds, cumulative release data were fitted to 3 widely used kinetic models for matrix-based delivery systems: zero-order, Higuchi, and Korsmeyer–Peppas. These models were selected because they capture the release patterns expected for ceramic scaffolds, namely constant-rate release (zero-order), diffusion-controlled release from a matrix (Higuchi), and semi-empirical classification of the dominant transport mechanism (Korsmeyer–Peppas). The zero-order model was expressed as *Q*_t_ = *Q*_0_ + *k*_0_*t*, the Higuchi model as *Q*_t_ = *k*_H_ *t*^(1/2)^ and the Korsmeyer–Peppas model as *Q*_t_/*Q*_∞_ = *k*_KP_ *t^n^*^KP^, where *Q*_t_ is the cumulative amount of DIPY released at time t, *Q*_0_ is the intercept of the zero order regression, *Q_∞_* is the total amount released at infinite time, and *k*_0_, *k*_H_ and *k*_KP_ are the corresponding release constants, with *n*_KP_ denoting the dimensionless release exponent. Because *Q*_t_/*Q*_∞_ is dimensionless and time was expressed in hours, *k*_KP_ has units of *h*^−*n*KP^. For porous scaffolds, fitting cumulative mass vs. time (zero-order model) yielded only moderate coefficients of determination (*R*^2^ = 0.604–0.629), indicating that drug release from porous scaffolds does not conform to zero-order kinetics. Plotting cumulative mass against the square root of time (Higuchi model) yielded higher *R*^2^ values (0.744–0.769), especially for the 250 µL group. The Higuchi rate constants also increased with coating volume. Korsmeyer–Peppas analysis was conducted for the porous scaffolds using log-log plots of fractional drug release versus time, considering both the entire release period and only the initial 60% of cumulative release. When the full release profile was fitted, the kinetic constant *k*_KP_ increased with coating volume, and the corresponding fits showed only moderate coefficients of determination (*R*^2^ = 0.813–0.849), while the release exponent *n*_KP_ decreased as coating volume increased (Table 2). In contrast, restricting the analysis to the first 60% of cumulative release markedly improved the goodness of fit, with *R^2^* values increasing to 0.943–0.966 at higher coating volumes, indicating that the Korsmeyer–Peppas model describes the early-stage release behavior substantially better than the later phases.

For solid scaffolds, linear fitting of cumulative mass vs. time (zero-order model) produced only moderate coefficients of determination (*R*^2^ = 0.497–0.682), indicating that solid scaffolds do not follow ideal zero-order release. The zero-order rate constants increased with coating volume, reflecting faster overall release from more heavily loaded scaffolds. Plotting cumulative mass released against √time (Higuchi model) produced fits that were comparable to or slightly better than zero-order (*R*^2^ = 0.644–0.816), with Higuchi rate constants increasing with coating volume for the full release period. Korsmeyer–Peppas fitting of the solid scaffolds showed that the kinetic constant *k*_KP_, accompanied by the corresponding *R^2^* (0.714–0.933) values, increased with increasing coating volume. Consistent with the porous scaffold behavior, the release exponent *n*_KP_ decreased as the coating volume increased (Table 3). When the analysis was restricted to the first 60% of cumulative release, the fit improved for the 500 and 1000 µL groups, with *R^2^* values of 0.983 and 0.984, respectively, whereas the 250 µL group showed a lower but still acceptable fit of 0.902.

### 3.4. MTT Assay

**Figure 3:**
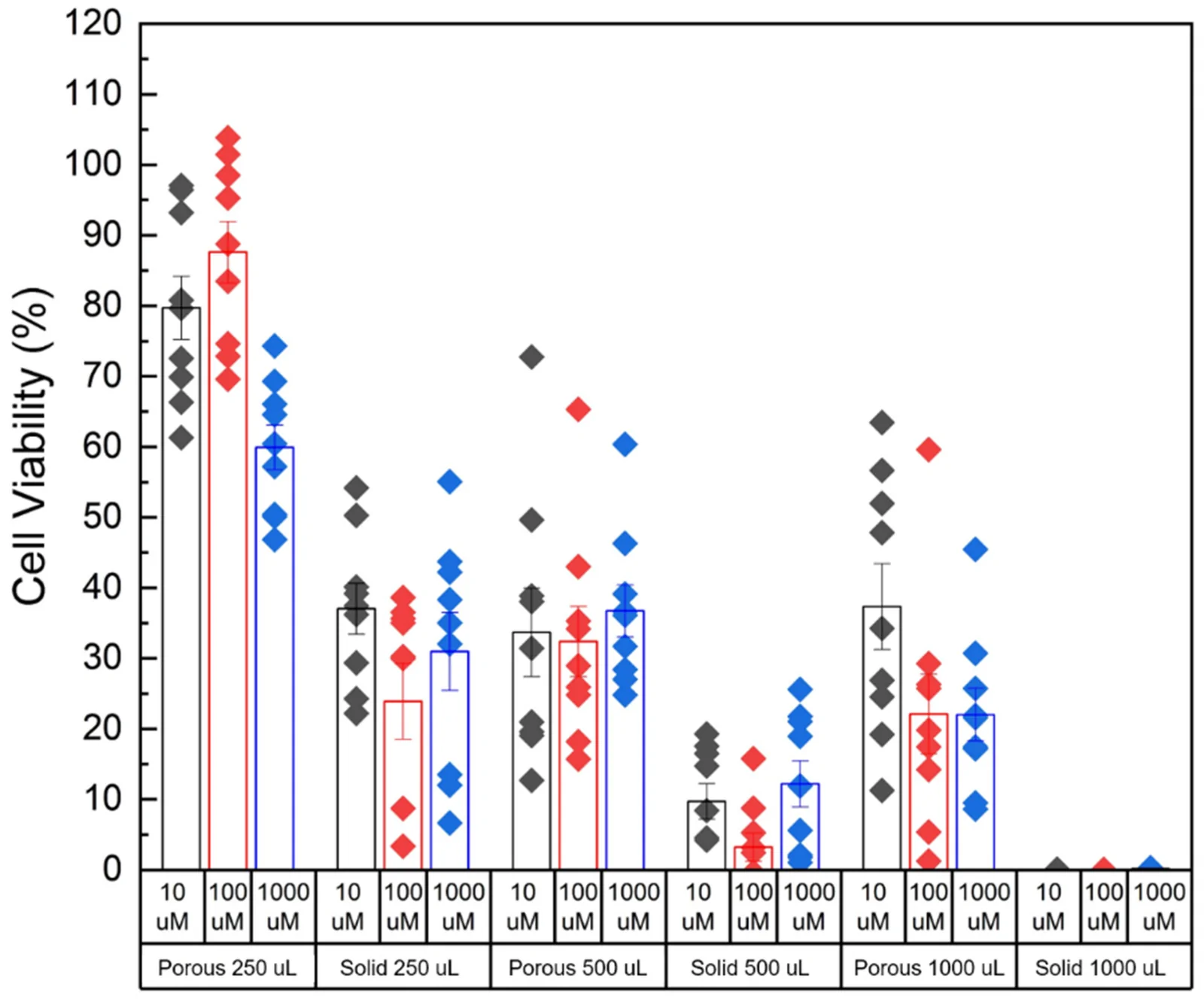
Cell viability assay for porous and solid β-TCP scaffolds coated with 3 volumes (250, 500, 1000 µL) of DIPY at 10, 100, or 1000 µM, with PBS-only vehicle as controls at each volume. Data are presented as mean ± standard error of the mean for *n* = 3 independent samples per group, with each scaffold measured in triplicate; after dissolution of formazan in DMSO, these individual absorbance readings (3 per scaffold) are shown as symbols.

For porous scaffolds, a significant main effect of coating volume on cell viability was observed (F(2,18) = 52.16, *p* < 0.001, partial *η*^2^ = 0.85), whereas the main effect of DIPY concentration was not significant (F(2,18) = 2.32, *p* = 0.127, partial *η*^2^ = 0.21). Post hoc tests showed that 250 µL coated porous scaffolds exhibited higher viability than both 500 µL and 1000 µL groups (*p* < 0.001), while 500 µL and 1000 µL did not differ significantly (*p* > 0.3). The volume and concentration interaction did not reach statistical significance (F(4,18) = 2.46, *p* = 0.083), indicating that the effect of coating volume on cellular viability was similar across concentrations (Figure 3). Similarly, for solid scaffolds, two-way ANOVA revealed a significant main effect of coating volume on cell viability (F(2,18) = 15.92, *p* < 0.001, partial *η*^2^ = 0.64), while the main effect of DIPY concentration was not significant (F(2,18) = 1.64, *p* = 0.223, partial *η^2^* = 0.15). Post hoc comparisons (Tukey HSD) indicated that 250 µL-coated solid scaffolds exhibited significantly higher cell viability relative to both 500 µL and 1000 µL cohorts (both *p* < 0.001), with no significant difference between the 500 µL and 1000 µL groups (*p* = 0.901). The concentration and volume interaction was not significant (F(4,18) = 0.27, *p* = 0.891), indicating that viability responses were consistent across concentrations regardless of coating volume.

### 3.5. Surface Characterization

**Figure 4:**
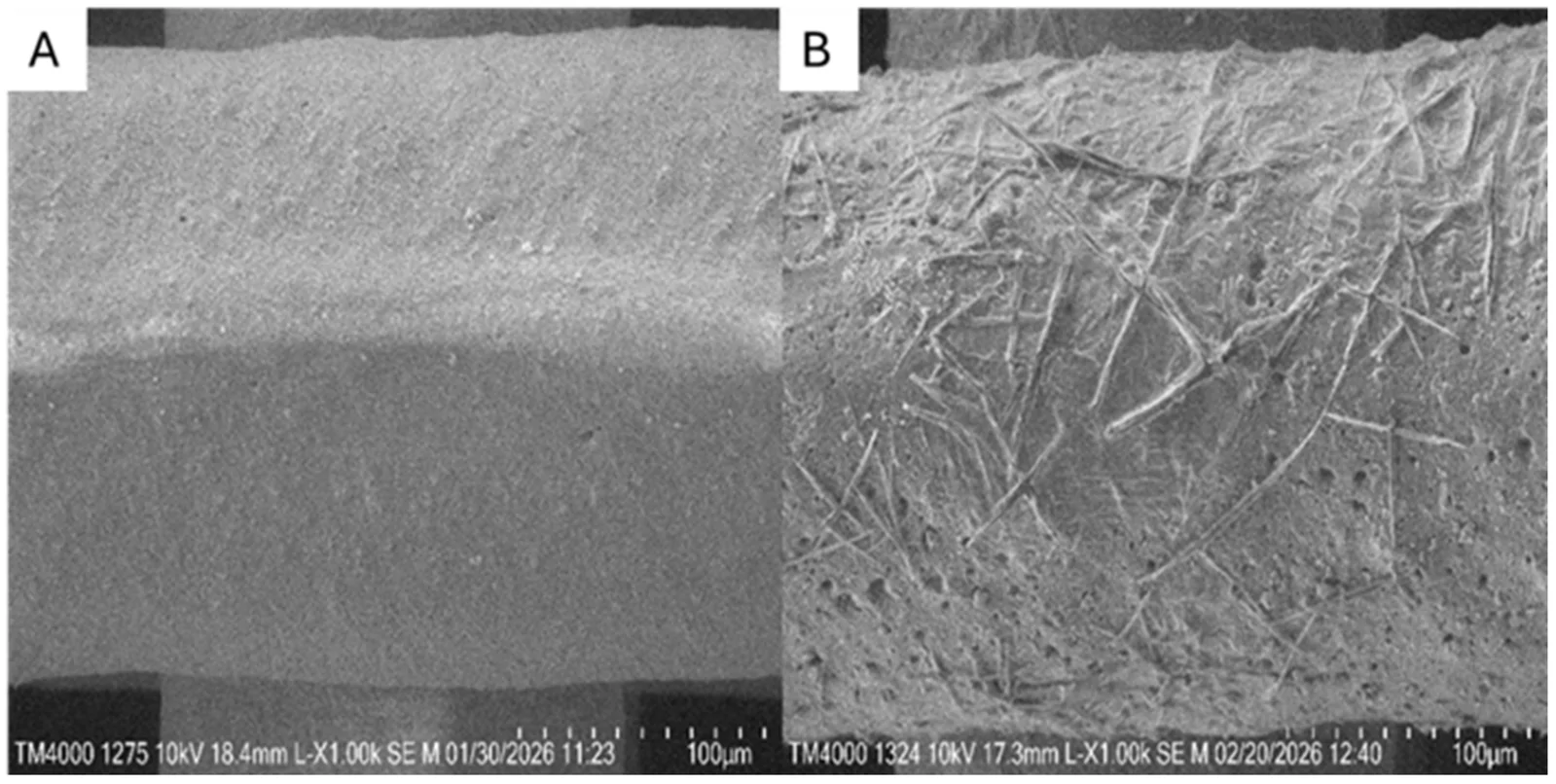
Representative SEM micrographs of the (**A**) uncoated and (**B**) coated β-TCP scaffolds.

**Figure 5:**
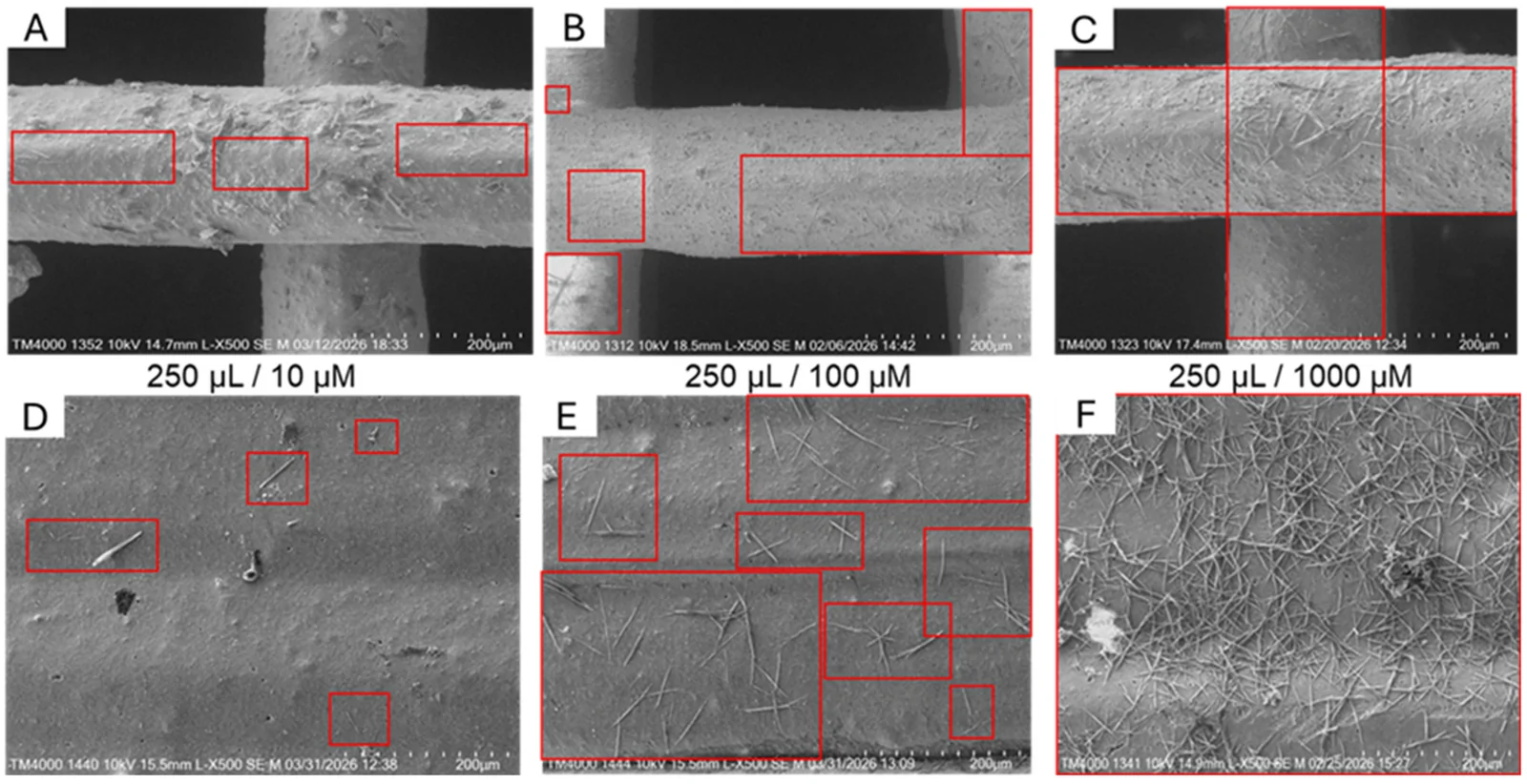
Representative SEM micrographs of the DIPY coated β-TCP scaffolds at a coating volume of 250 µL: (**A**) porous at a 10 µM DIPY concentration, (**B**) porous at a 100 µM DIPY concentration, (**C**) porous at a 1000 µM DIPY concentration, (**D**) solid at a 10 µM DIPY concentration, (**E**) solid at a 100 µM DIPY concentration, and (**F**) solid at a 1000 µM DIPY concentration. Red boxes highlight the presence of the elongated, needle-shaped crystal-like structures. In the porous 10 µM group, some bright protrusions correspond to β-TCP surface projections rather than DIPY crystals, whereas DIPY crystals appear as thin, elongated deposits.

SEM imaging of uncoated β-TCP scaffolds revealed the native ceramic surface morphology (Figure 4A). Upon DIPY coating, elongated, needle-shaped crystal-like structures were observed on the scaffold surface (Figure 4B). The presence of these crystal structures was concentration-dependent for both porous and solid architectures. In brief, the 10 µM group showed sparse, isolated crystals; the 100 µM group exhibited moderately increased coverage; and at 1000 µM, the crystals covered nearly the entire scaffold surface (Figure 5A–F).

**Figure 6:**
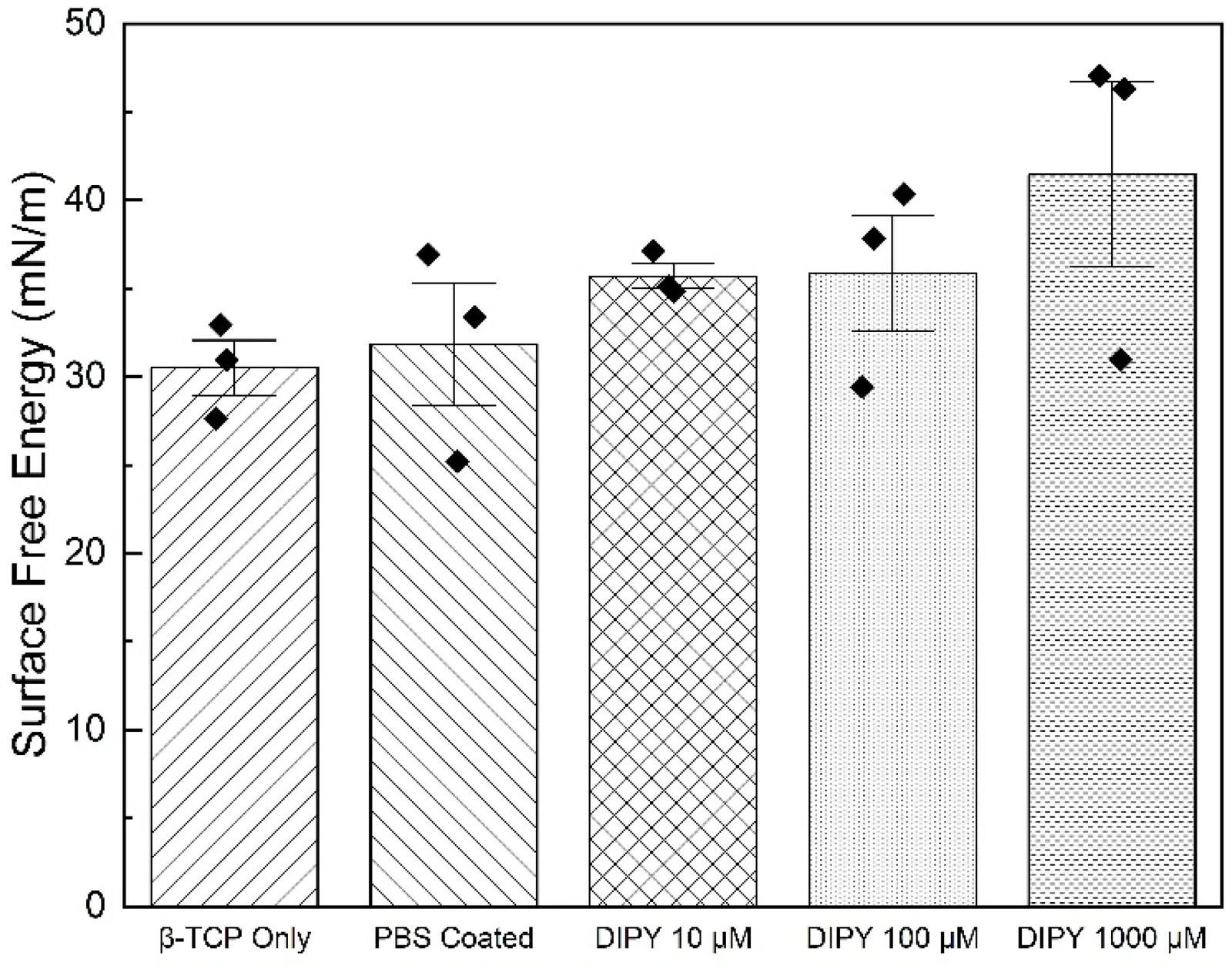
Surface free energy of β-TCP (mN/m) solid scaffolds after PBS or DIPY coating presented as mean ± standard error of the mean for *n* = 3 independent solid β-TCP scaffolds per group. Symbols overlaying each bar indicate the individual surface free energy values for each scaffold.

Contact angle-derived surface energy values were similar across all groups (10–1000 µM). No significant effect of coating was observed on mean surface energy values (F(4,10) = 1.71, *p* = 0.224). Although Levene’s test based on the mean indicated heterogeneity of variances across groups (*p* = 0.044), a variance-robust Welch ANOVA did not detect significant differences (Welch F(4,4.44) = 2.0, *p* = 0.245) (Figure 6).

## 4. Discussion

This study examined the physicochemical and bioactive properties of DIPY-loaded 3D-printed β-TCP scaffolds, comparing solid and porous architectures across a range of coating concentrations and volumes. The porous β-TCP scaffold architecture was selected to fall within geometries previously shown to support cancellous bone-like mechanical performance. Macroporous β-TCP scaffolds with interconnected 400–550 µm pores at ~65% porosity have achieved compressive strengths of ~10 MPa [41], while graded calcium phosphate scaffolds combining an inner ~500 µm macroporous region with a compact outer shell have reached ~36 MPa at ~56% porosity, mimicking cortico-cancellous bone [42]. From a design standpoint, the porous β-TCP scaffolds employed a rectilinear lattice of cylindrical struts arranged orthogonally to generate interconnected square pores. The selection of strut geometry is an important consideration in the design of additively manufactured lattices for mechanically demanding applications, including load-bearing implants, as strut cross-sectional shape can influence printing accuracy and mechanical response [43].

The selected coating range (10, 100, and 1000 µM) was chosen to correspond to concentrations with established in vivo osteogenic activity [18,28] alongside evidence that higher doses yield a plateau in bone formation, consistent with receptor-saturation pharmacodynamics [30]. The 10 µM condition was included to investigate the lower boundary of effective loading, which is lower than that investigated in previous studies. Previous studies utilizing DIPY have used collagen-mediated immersion methods to coat scaffolds [18,28,30]. In contrast, this study used a custom-fabricated coating technique for solid scaffolds, confining drug deposition to specific external surfaces. This enhanced dose control on non-porous substrates and allowed for direct calculation of theoretical drug mass. Surface-restricted strategies for β-TCP scaffolds include dropwise pipetting with defined loadings of 200–500 µg [37] and vacuum-assisted delivery of controlled coating volumes [44]. Cumulative drug release data are reported as absolute mass (µg) rather than percentage of the loaded dose, as the drug loading was not experimentally verified through extraction assays, and expressing release as a percentage would assume quantitative, loss-free deposition, an assumption that cannot be confirmed without protocols such as those used by Jang et al. [45]. After PBS background subtraction, only the 1000 µM coating showed detectable cumulative release, while the 10 and 100 µM groups remained below the assay’s detection limit. UV–Vis spectrophotometry generally exhibits a lower limit of quantification for small molecules of 1–2 µg/mL [46]. Given the low theoretical drug masses at 10 and 100 µM (approximately 1.26–50.46 µg per scaffold), it is likely that DIPY was released but remained below the spectrophotometric detection threshold under the present assay conditions. Furthermore, the calibration-based validation indicated a Limit of Detection (LOD) of 73.49 µM and a Limit of Quantification (LOQ) of 222.69 µM for DIPY. Consequently, the 10 µM loading condition lies below the detection limit of the assay, and the 100 µM condition lies between LOD and LOQ, where signals are not reliably quantifiable. The minimal or undetectable DIPY signal for these groups can therefore be attributed to analytical sensitivity constraints rather than the complete absence of drug release. In addition, calcium phosphate precipitation and dissolution in PBS-based media can affect absorbance baselines by introducing ionic interference, which is especially problematic at early time points and further complicates the detection of low-level release [47,48].

Release profiles over time depended on the amount of DIPY loaded (as controlled by coating volume). At 1000 µM, both porous and solid scaffolds showed a biphasic DIPY release profile, with a clear burst in the early time points followed by a slower, sustained increase in cumulative release. This pattern may be a result of rapid loss of drug located near the surface and more gradual release from regions deeper within the coating or scaffold. Solid scaffolds released more DIPY overall than porous scaffolds at the same nominal loading, which is likely related to their non-porous geometry: on solid constructs, DIPY is mainly confined to the external faces that are directly exposed to the release medium, whereas in porous scaffolds part of the applied drug likely diffuses into less accessible parts of the pore network and does not fully elute under the conditions used here. In this context, increasing coating volume at 1000 µM mainly increased the total amount of DIPY delivered, rather than altering the general shape of the release curve, with solid scaffolds providing the highest cumulative dose. Since 1000 µM DIPY has previously been reported to enhance osteogenic regeneration without adverse effects, the observed combination of an initial burst followed by prolonged, lower-level release in these scaffolds appears compatible with exposure profiles that may support a sustained osteogenic response, although the present findings do not permit direct comparison of the in vivo efficacy of the porous and solid architectures [16].

All solid scaffolds were coated bilaterally, with half of the total volume applied to each face (125, 250, or 500 µL per face for the 250, 500, and 1000 µL groups, respectively). Thus, the local coating volume per face increased from 125 to 250 to 500 µL as total volume rose from 250 to 500 to 1000 µL, respectively. Despite this, the 250 and 500 µL groups showed very similar cumulative release, and the ANOVA did not detect a significant volume effect between these two doses. A plausible explanation is that, within this intermediate range, increasing the local coating thickness per face does not proportionally increase the fraction of DIPY that can diffuse into the release medium under the present test conditions, i.e., additional drug may be present in deeper layers of the surface deposit but contribute little to the measured cumulative release. In contrast, the 1000 µL coating provides sufficient excess to produce a clear increase in released mass.

Examination of the kinetic parameters clarifies how coating volume and scaffold architecture influence DIPY release. In both porous and solid scaffolds, the zero-order rate constants and Higuchi constants increased with coating volume, indicating that more heavily loaded scaffolds released more DIPY per unit time and exhibited faster diffusion-controlled release from the ceramic matrix. The Korsmeyer–Peppas constant likewise rose with increasing volume, consistent with higher fractional release rates as more DIPY was available on accessible surfaces, whereas the Korsmeyer–Peppas exponent remained low (well below 0.5) and decreased slightly with higher volumes, supporting a predominantly Fickian, diffusion-driven process rather than erosion-dominated or ideal zero-order release.

Across both architectures, the Higuchi model provided a better fit than zero-order kinetics, in line with surface-associated, diffusion-controlled release and prior reports of burst-then-sustained behavior in β-TCP and related calcium phosphate systems [37,40,44]. Korsmeyer–Peppas analysis further indicated that the initial ~60% of release was reasonably approximated by a power-law relationship, whereas the full biphasic profile could not be fully captured by a single empirical model. Taken together, these findings suggest that increasing coating volume at 1000 µM primarily augments the diffusion-mediated release rate and total delivered dose, while the underlying release mechanism remains diffusion-controlled, and that differences between porous and solid β-TCP scaffolds mainly reflect how much of the loaded DIPY is exposed to the release medium rather than a fundamental change in release mode.

For the drug release tests, the ANOVA models were estimated with Greenhouse–Geisser corrections to address sphericity violations. This reduces the effective degrees of freedom and yields more conservative tests. Given the sample size (*n* = 3 per group), the non-significant coating volume effects observed in solid scaffolds can be best interpreted as indicating that any volume-dependent differences, if present, are unlikely to be large under the conditions tested.

SEM analysis revealed needle-shaped DIPY crystals on scaffold surfaces. Surface coverage increased from sparse at 10 µM to nearly continuous at 1000 µM. The morphology of these needles showed DIPY crystallization during solvent evaporation. The absence of similar features on uncoated controls, as well as the clear concentration dependency, suggests that these needles are drug-specific rather than apatite-derived [49]. The contact angle and surface energy measurements were conducted only on solid scaffold specimens, as the rough, highly porous architecture of the porous scaffolds did not allow reproducible droplet formation or reliable contact angle determination on their surfaces. Moreover, the surface energy measurements did not differ significantly among uncoated, PBS-coated, and DIPY-coated scaffolds. This is consistent with the intrinsically high-energy, hydrophilic nature of sintered β-TCP relative to the restricted area coverage expected from a thin drop-cast surface coating [50]. Improved hydrophilicity with DIPY has been observed only at considerably higher bulk loadings (e.g., 10% w/w in thermoplastic polyurethane), well beyond the surface-coating levels used in the present study [51]. Nonetheless, from a practical standpoint, the absence of a significant change is advantageous, indicating that DIPY coating does not render the β-TCP surface more hydrophobic and thus preserves its cell-adhesive wettability.

The 24 h direct MTT assay was employed as an initial assessment of acute cytocompatibility and early cell attachment on DIPY-coated β-TCP scaffolds, consistent with the overall focus on physicochemical characterization and short-term biological screening of coating parameters. In this context, the 24 h MTT assay served as a literature-supported method for short-term cytotoxicity screening of β-TCP and other resorbable ceramic scaffolds, enabling direct comparison of early cell responses to varying DIPY concentrations and coating volumes under standardized conditions [52,53]. No statistically significant differences in cell viability was observed among the different DIPY coating concentrations. Increasing coating volume, however, was associated with a marked reduction in viability across both scaffold types, particularly at 500 and 1000 µL. A likely contributor to this effect is residual DMSO from the coating process, as DMSO has been reported to influence cell viability in a concentration- and exposure-dependent manner, with cytotoxic effects becoming more apparent as residual levels increase [54]. Based on these values, the 24 h data indicates that the 250 µL coating conditions for both scaffold types maintain comparatively higher viability in this acute, direct-contact assay, whereas the higher-volume conditions exhibit reduced viability and may have cytotoxic potential in the same test setting. This suggests that the drug itself may be tolerated at the concentrations used but that coating volume is a critical variable. Notably, porous scaffolds maintained higher viability than solid scaffolds overall, suggesting that the interconnected porous architecture may have provided a more supportive cellular microenvironment, potentially through enhanced nutrient transport, improved waste removal, and greater available surface area for cell attachment.

Among the limitations of this study, UV–Vis spectrophotometry lacked sensitivity to quantify release from 10 and 100 µM groups. Future studies should focus on higher-sensitivity techniques such as high-performance liquid chromatography (HPLC) with fluorescence detection or liquid-core waveguide UV, which offer considerably lower detection limits [46]. Since the porous scaffolds were immersed rather than surface-coated, actual drug loading may differ from theoretical estimates. Accurate determination of the DIPY content in each scaffold, therefore, requires solvent extraction followed by HPLC analysis. Future studies should also include quantitative analysis of pore size and morphology before and after DIPY coating to determine whether drug crystal deposition measurably modifies pore architecture in ways that could affect bone ingrowth and nutrient transport.

Based on the 24 h cell viability results, the 250 µL coating volume appeared to be the most suitable screening volume among the conditions tested in this study. However, because this short-term time point primarily reflects initial cell attachment and acute cytocompatibility rather than longer-term osteoblast function, inclusion of extended proliferation, ALP activity, and osteogenic differentiation assays would likely strengthen support for its functional superiority. Furthermore, all release studies were carried out under static conditions, which do not simulate the dynamic interstitial fluid flow found in vivo implant sites and may hence underestimate true release rates [47]. Therefore, in vivo studies in relevant bone defect models are still required to determine whether the physicochemical framework identified here translates into improved bone regeneration.

## 5. Conclusions

Critical-sized bone defects require scaffolds with predictable drug release, and this study provides key physicochemical data for DIPY-loaded 3D-printed β-TCP scaffolds in solid and porous forms. At 1000 µM, both scaffold types exhibited biphasic, diffusion-dominated release that was only partially captured by standard kinetic models. Porous scaffolds showed strong volume-dependent release due to drug penetration into the macropore network, whereas solid scaffolds showed volume-independent, surface-limited release. Coating volume had a greater effect on viability than drug concentration; within this context, 250 µL yielded the highest cell viability, but cannot yet be defined as an optimal loading volume. Together, these results highlight key variables to be refined in future long-term in vivo studies.:

## Figures and Tables

**Table 1:** Theoretical drug mass loaded per scaffold across all coating conditions.

Loading Concentration (µM)	Loading Volume (µL)	Total Moles (nmol)	Drug Loading Content (µg/mm^2^)	Total Drug Mass (µg)
10	250	2.5	0.009	1.26
	500	5	0.019	2.52
	1000	10	0.038	5.05
100	250	25	0.095	12.62
	500	50	0.190	25.23
	1000	100	0.380	50.46
1000	250	250	0.949	126.16
	500	500	1.898	252.32
	1000	1000	3.795	504.63

**Table 2:** Zero-order, Higuchi, and Korsmeyer–Peppas kinetic parameters for DIPY release from porous β-TCP scaffolds coated with 1000 µM DIPY at the three coating volumes (250, 500, and 1000 µL). Rate constants: *k*_0_ (zero-order), *k*_H_ (Higuchi), *k*_KP_ (Korsmeyer–Peppas; *h*^−*n*KP^) together with the release exponent *n*_KP,_ are obtained by linear regression of: cumulative mass released vs. time (zero-order) or cumulative mass vs. √time (Higuchi) or log-transformed fractional release (log (*Q*_t_/*Q*_∞_)) vs. log (*t*) for the full release profile (Korsmeyer–Peppas). The corresponding coefficients of determination (*R*^2^) are shown for each model and coating volume.

Coating Volume (µL)	*k*_0_ (µg/h)	*R*^2^ (Zero-Order)	*k*_H_ (µg/√h)	*R*^2^ (Higuchi)	*k*_KP_ (*h*^−*n*KP^): Full Profile	*R*^2^ (Korsmeyer–Peppas: Full Profile)	*n*_KP_ (Full Profile)
250	0.26	0.629	6.65	0.769	0.23	0.813	0.26
500	0.31	0.611	7.98	0.754	0.33	0.829	0.20
1000	0.38	0.604	9.70	0.744	0.41	0.849	0.16

**Table 3:** Zero-order, Higuchi, and Korsmeyer–Peppas kinetic parameters for DIPY release from solid β-TCP scaffolds coated with 1000 µM DIPY at three coating volumes (250, 500, and 1000 µL). Rate constants: *k*_0_ (zero-order), *k*_H_ (Higuchi), *k*_KP_ (Korsmeyer–Peppas; *h*^−*n*KP^) together with the release exponent *n*_KP_, are obtained by linear regression of: cumulative mass released vs. time (zero-order) or cumulative mass vs. √time (Higuchi) or log-transformed fractional release (log (*Q*_t_/*Q*_∞_)) vs. log (*t*) for the full release profile (Korsmeyer–Peppas). The corresponding coefficients of determination (*R*^2^) are shown for each model and coating volume.

Coating Volume (µL)	*k*_0_ (µg/h)	*R*^2^ (Zero-Order)	*k*_H_ (µg/√h)	*R*^2^ (Higuchi)	*k*_KP_ (*h*^−*n*KP^): Full Profile	*R*^2^ (Korsmeyer–Peppas: Full Profile)	*n*_KP_ (Full Profile)
250	0.28	0.682	7.09	0.816	0.42	0.714	0.16
500	0.32	0.522	8.44	0.658	0.52	0.810	0.12
1000	0.38	0.497	10.00	0.644	0.55	0.933	0.10

## Data Availability

The original contributions presented in the study are included in the article/Supplementary Materials, further inquiries can be directed to the corresponding author.

## Supplementary Materials

The following supporting information can be downloaded at: https://www.mdpi.com/article/10.3390/jfb17080396/s1, Figure S1: Standard curve generated from serial dilutions of dipyridamole (DIPY). Reference [55] is cited in the supplementary materials.
