# Supplementary material for: Dipyridamole-Coated 3D-Printed β-Tricalcium Phosphate Scaffolds: Spectrophotometric Characterization, Drug Release Kinetics, and In Vitro Evaluation to Guide Critical-Sized Bone Defect Repair Studies
Source: J Funct Biomater. 2026 Aug 11;17(8):396. doi: 10.3390/jfb17080396 (PMC13514228; doi:10.3390/jfb17080396)
Supplement: Supplementary file 1 [file jfb-17-00396-s001.zip › jfb-4442683-supplementary.pdf]

Supplementary Figures

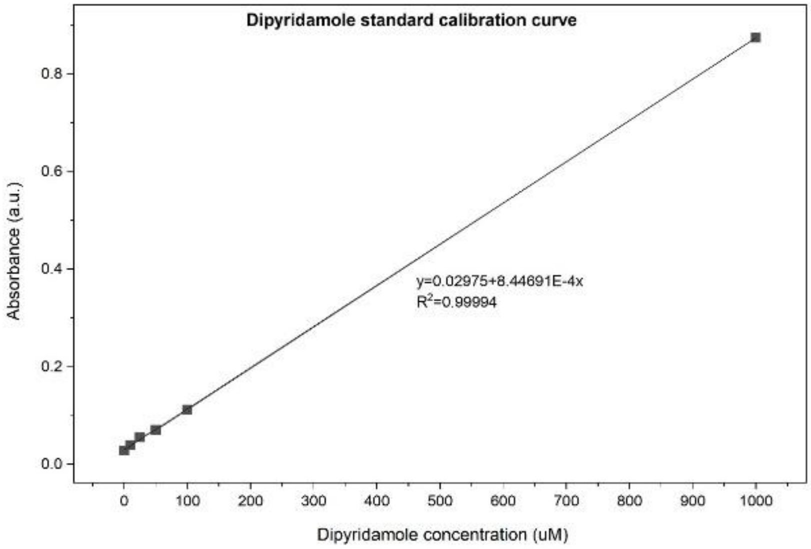

Supplementary Figure 1: Standard curve generated from serial dilutions of dipyridamole (DIPY). Standard curve shows a linear relationship between absorbance at 405 nm and drug concentration over the calibrated range ($R^2 = 0.9999$). Limits of detection (LOD) and quantification (LOQ) were calculated from the calibration curve according to the ICH Q2 guideline [55], using LOD = 3.3σ/S and LOQ = 10σ/S, where σ is the residual standard deviation of the regression, and S is the slope of the calibration line. This yielded a LOD of 73.49 μM and a LOQ of 222.69 μM.
